# Gene polymorphisms of VEGF and KDR are associated with initial fast peritoneal solute transfer rate in peritoneal dialysis

**DOI:** 10.1186/s12882-022-02975-5

**Published:** 2022-11-14

**Authors:** Yue Qian, Li Ding, Liou Cao, Zanzhe Yu, Xinghua Shao, Ling Wang, Minfang Zhang, Qin Wang, Xiajing Che, Na Jiang, Hao Yan, Wei Fang, Yan Jin, Jiaying Huang, Aiping Gu, Zhaohui Ni

**Affiliations:** Department of Nephrology, Renji Hospital, School of Medicine, Shanghai Jiao Tong University, Shanghai, China

**Keywords:** Peritoneal dialysis, VEGF, KDR, Gene polymorphisms, SNP, Peritoneal solute transfer rate

## Abstract

**Background:**

Peritoneal dialysis (PD) is an effective and successful renal replacement therapy. The baseline peritoneal solute transfer rate (PSTR) is related to local membrane inflammation and may be partially genetically determined. Herein, we focused on vascular endothelial growth factor (VEGF) and its receptor, kinase insert domain containing receptor (KDR).

**Methods:**

This study recruited 200 PD patients from Renji Hospital in Shanghai, China. We analysed the association between the polymorphisms of VEGF and KDR and the 4-hour dialysate-to-plasma ratio for creatinine (4 h D/P Cr), which was measured between one and three months after initiating PD.

**Results:**

The CC genotype in VEGF rs3025039 and the AA genotype in KDR rs2071559 were both positively associated with a fast baseline PSTR (VEGF rs3025039 CC vs. TT + TC: 0.65 ± 0.12 vs. 0.61 ± 0.11; P = 0.029; KDR rs2071559 AA vs. GA + GG: 0.65 ± 0.12 vs. 0.62 ± 0.12; P = 0.039).

**Conclusion:**

Baseline PSTR was partly determined by VEGF and KDR gene polymorphisms.

## Background

Peritoneal dialysis (PD) is an effective and important treatment for renal replacement in patients with end-stage renal disease (ESRD). Studies from all regions of the world have shown that faster baseline peritoneal solute transfer rate (PSTR) is related to higher risk for technology failure and death [1–4].

The characteristics of peritoneal baseline transport depend on the structure and function of the pre-dialysis peritoneum, which is related to race, age, sex, and underlying disease [5]. However, only 5–11% of the total interindividual variability in the PSTR can be explained by these demographic or clinical variables. Local peritoneal membrane inflammation seems to play an important role in this process. Dialysate IL-6 is the marker with the strongest known association with PSTR, whereas systemic inflammation is associated with comorbidity and patient survival [6–8]. Evidence from several small single-centre studies shows that some of the between-patient variation may be accounted for by genetic factors related to proinflammatory factors [9, 10].

Neovascularization contributes to both initial fast PSTR and fast PSTR in long -term PD. Although more studies have focused on neovascularization in long-term exposure in PD, several studies have shown higher concentrations of VEGF and KDR in peritoneal tissue in PD patients with initial high/high average transport than in patients with low/low average transport, which means that local expression of VEGF and KDR in peritoneal tissue can affect peritoneal baseline transport by increasing the number of new peritoneal vessels and increasing inflammation status [11, 12].

Vascular endothelial growth factor (VEGF) and its main receptor vascular endothelial growth factor receptor-2 (VEGFR2, or kinase insert domain containing receptor, KDR) are key factors involved in angiogenesis and inflammation [13]. Single-nucleotide polymorphisms (SNPs) mainly refer to polymorphisms of DNA sequences caused by variations in single nucleotides at the genome level, which may affect the function of proteins and lead to disease. SNPs of VEGF and KDR have been reported to be related to many diseases, including tumours, by participating in angiogenesis [14–21].

Therefore, we speculate that SNPs may affect the expression of VEGF and KDR in peritoneal tissue at the gene level, thus causing differences in the baseline PSTR by affecting the number of blood vessels and the inflammatory state.

The aim of this study was to investigate the genetic association between VEGF and KDR gene polymorphisms and the type of baseline PSTR in PD patients and to try to find a reliable genetic locus that can predict initial high peritoneal transport, thereby revealing the characteristics of peritoneal transfer in the early stage.

## Methods

### Clinical characteristics of the study population

In this study, a total of 200 patients starting PD from January 1, 2004, to January 31, 2014, in the Department of Nephrology, Renji Hospital, Shanghai Jiaotong University, School of Medicine were included.

The inclusion criteria were as follows:

(1) Han Chinese;

(2) PD started within 3 months after catheter implantation;

(3) data available from the first peritoneal equilibration test (PET) between 1 and 3 months of starting PD;

(4) agreed to participate in the study.

Patients who were on long-term haemodialysis or who underwent transplantation before the current PD episode were excluded.

### Measurement of peritoneal transfer rate

Classic 2.5% PET was performed between 1 and 3 months after PD initiation in all patients. The primary results were expressed as the 4-hour dialysate-to-plasma ratio for creatinine (4 h D/P Cr).

According to the 4 h D/P Cr, patients were classified into four transport types: high transport status (H, 4 h D/P Cr > 0.8), high average transport status (HA, 4 h D/P Cr 0.65–0.8), low average transport status (LA, 4 h D/P Cr 0.5–0.64), and low transport status (4 h D/P Cr < 0.5). In this study, we divided the patients into two groups: the H/HA transporter group and the L/LA transporter group.

### SNP selection

**Fig. 1:**
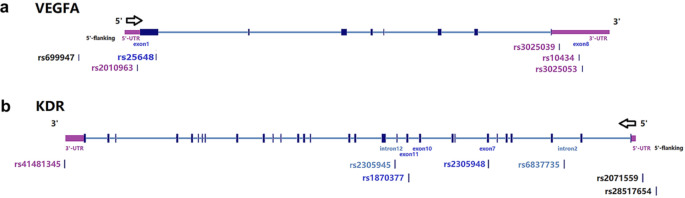
Gene location of tag-SNPs in VEGF and KDR

Six SNPs of VEGF and seven SNPs of KDR were obtained from International HapMap Project Databases (Fig. 1; Table 1). The screening conditions and scope were as follows: Han Chinese in Beijing & Southern Han Chinese (CHB&CHS); the gene was amplified by 2000 bp upstream and 1000 bp downstream; minor allele frequency (MAF) > 0.05; r^2^ > 0.8; there was no linkage disequilibrium between each other. We used the Hardy Weinberg equilibrium (HWE) test for all the alleles in all samples as well as each group. If the P value < = 0.001, we considered the allele was not in conformity with the HWE in this population and would not use it for further analysis.

**Table 1:** Selected SNPs of VEGF and KDR

Gene	SNPs	Chr Position	Gene Region
VEGF	rs10434	Chr6:43,753,212	UTR 3
	rs2010963	Chr6:43,738,350	UTR 5
	rs25648	Chr6:43,738,977	Exon 1
	rs3025039	Chr6:43,752,536	UTR 3
	rs3025053	Chr6:43,753,325	UTR 3
	rs699947	Chr6:43,736,389	upstream
KDR	rs1870377	Chr4:55,972,974	Exon 11
	rs2071559	Chr4:55,992,366	upstream
	rs2305945	Chr4:55,971,846	Intron 12
	rs2305948	Chr4:55,979,558	Exon 7
	rs28517654	Chr4:55,993,468	upstream
	rs41481345	Chr4:55,944,618	UTR 3
	rs6837735	Chr4:55,985,815	Intron 2

### SNP genotyping

Venous blood was collected at the time of PD initiation. DNA was extracted according to the standard process using Wizard® Genomic DNA Purification Kit. The SNPs of VEGF and VEGFR2 were genotyped by a single-base primer extension assay. The sequences of the primers were shown in Table 2. The genomic DNA flanking the SNP was amplified by standard polymerase chain reaction (PCR) using forward and reverse primer pairs. The PCR machine was MJ Research PT-100, and the ABI PRISM® SNaPshot™ Multiplex Kit was used.

**Table 2:** Sequences of the primers of PCR

Gene	SNPs	Forward Sequences	Reverse Sequences
VEGF	rs10434	CTTCGCTTACTCTCACCTGCTTCTGA	GGATCCTGCCCTGTCTCTCTGTG
	rs2010963	ACGGCTTGGGGAGATTGCTCTA	CCCCAAAAGCAGGTCACTCACT
	rs25648	GGGCCGGGGAGGAAGAGTAG	CAATGCACCCAAGACAGCAGAA
	rs3025039	CCACACCATCACCATCGACAGA	ATCTTCCGGGCTCGGTGATTTA
	rs3025053	CTTCGCTTACTCTCACCTGCTTCTGA	GGATCCTGCCCTGTCTCTCTGTG
	rs699947	GTGCTGAGGATGGGGCTGACTA	AGGGAACAAAGTTGGGGCTCTG
KDR	rs1870377	CCTCCCTGGAAGTCCTCCACAC	CAGAATAGCTGCTTCCCTCCTGTATC
	rs2071559	CACAAGGGAGAAGCGGATACTCAG	CTTGGGGCTAGGCAGGTCACTT
	rs2305945	CACTGACTTCACATAAGCCCAGGAG	TCTGGAGGTTTGGGTTGGATCA
	rs2305948	TGGACCCTGACAAATGTGCTGTT	TGAGATGAAGAAATTTTTGAGCACCTT
	rs28517654	CCCTGCCCAGCCTTCACTTT	CCTCCCCAAATAAATACCTCCCAGAT
	rs41481345	AGCCACCCCCTCTTCCATTTTA	GCATAACAAAGGTCATAATGCTTTCAGC
	rs6837735	AAGAATTTTGCAGGAGGTGGTCTTG	TGGTTTCCTGGCTGTTCCCTTA

### Statistical analysis

The data were analysed by SPSS 25 and Prism 9. Categorical data are presented as the frequency (percentage); normally distributed continuous data are presented as the mean ± SD, and nonnormally distributed continuous data are expressed as the median (interquartile space). T tests and one-way ANOVA were used to analyse and compare the normally distributed data, and the Wilcoxon rank sum test was used to analyse and compare the nonnormally distributed data. The composition ratio of counting data was analysed and compared by the chi-square test. P < 0.05 was considered statistically significant.

## Results

### Clinical characteristics of the PD patients

According to the 4 h D/P Cr of their first PET, 94 patients had high/high average transporters, while 104 had low/low average transporters. The baseline clinical characteristics of the patients were shown in Table 3. H/HA transporters had lower haemoglobin (97.95 ± 21.48 vs. 106.21 ± 20.74, P = 0.006), serum albumin (34.55 (31.10, 38.80) vs. 36.49 (33.88, 40.10)) and ultrafiltration (-41.79 ± 595.13 vs. 227.89 ± 525.81) than L/LA group transporters. However, there were no significant differences in sex, age, BMI, underlying diseases (diabetes, hypertension), CRP, urine, urea clearance index (Kt/V) or normalized protein catabolic rate (nPCR) between the two groups.

**Table 3:** Clinical characteristics of the 200 PD patients

	H/HA (n = 94)	L/LA (n = 106)	P Value
Male (%)	56(59.6)	50(47.2)	0.079
Age (year)	51.47 ± 14.24	52.72 ± 14.78	0.545
BMI (kg/m^2^)	21.60(19.54, 23.63)	21.81(19.55, 23.73)	0.930
Diabetes (%)	20(21.3)	16(15.1)	0.274
Hb (g/L)	97.95 ± 21.48	106.21 ± 20.74	***0.006***
Alb (g/L)	34.55(31.10, 38.80)	36.49(33.88, 40.10)	***0.022***
CRP (mg/L)	4.97(1.00, 4.07)	6.47(0.66, 4.12)	0.697
UF (ml)	-41.79 ± 595.13	227.89 ± 525.81	0.001
Urine (ml)	1152.98 ± 686.61	1008.41 ± 593.31	0.112
4 h D/P Cr	0.74(0.69, 0.77)	0.54(0.50, 0.60)	***<0.001***
Kt/V	2.20(1.83, 2.53)	2.24(1.81, 2.54)	0.737
nPCR [g/(kg·d)]	0.95(0.77, 1.10)	1.06(0.78, 1.14)	0.655

BMI: body mass index; Hb: haemoglobin; Alb: serum albumin; CRP: C reactive protein; UF: ultrafiltration; 4 h D/P Cr: 4-hour dialysate over plasma ratio for creatinine; Kt/V: urea clearance index; nPCR: normalized protein catabolic rate

### Association between VEGF polymorphisms and PSTR

**Fig. 2:**
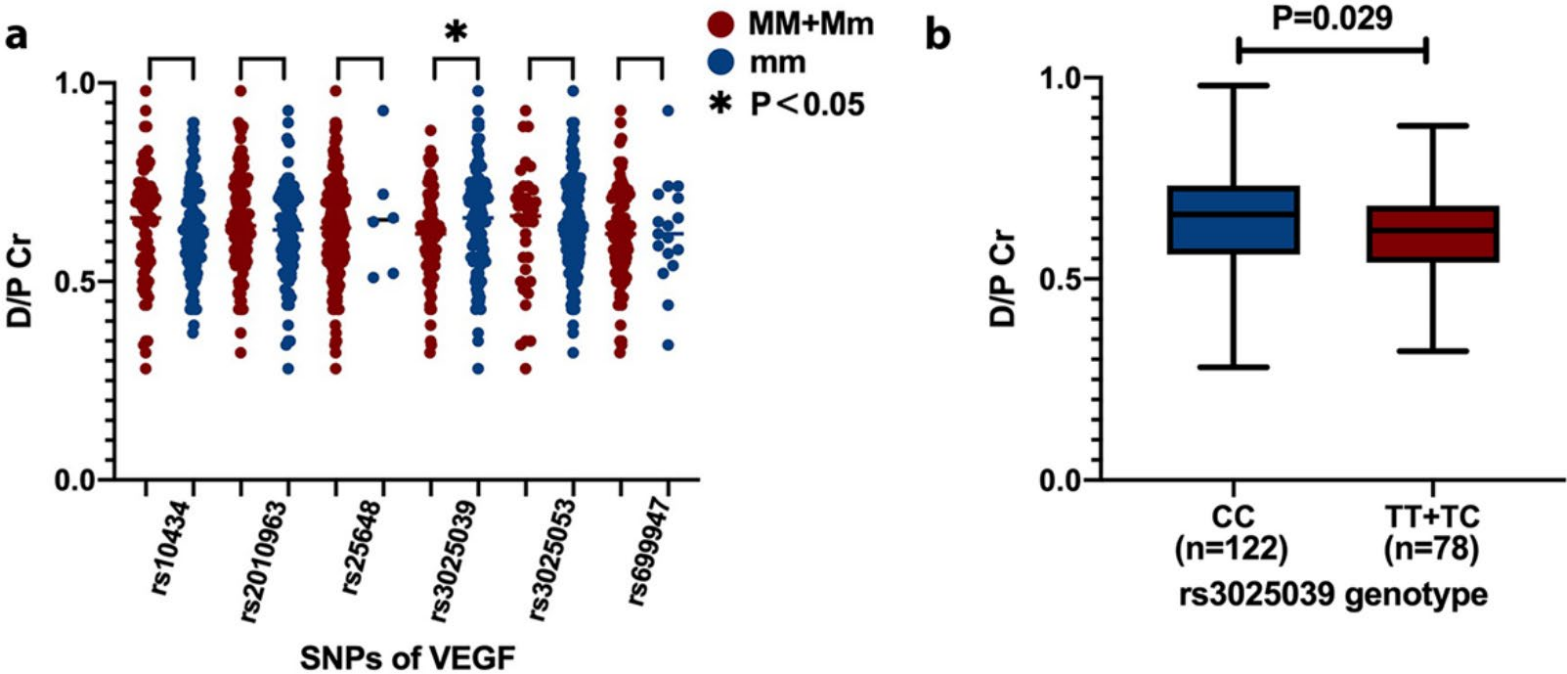
(a) VEGF polymorphisms and D/P Cr. M: alteration allele; m: reference allele.(b) *rs3025239 genotypes and D/P Cr*

The frequency and distributions of genotypes in VEGF are shown in Fig. 2a. The allelic distributions were all in conformity with Hardy-Weinberg equilibrium.

As shown in Fig. 2a, there was no significant association between D/P Cr and VEGF polymorphisms in rs10434, rs2010963, rs25648, rs3025053 and rs699947. VEGF SNPs in rs3025039 were significantly associated with PSTR.

In the rs3025039 polymorphism (Fig. 2b), patients with the CC genotype were related to higher D/P Cr (CC vs. TT + TC: 0.65 ± 0.12 vs. 0.61 ± 0.11; P = 0.029).

Moreover, we found that CC carriers had an increased H/HA transport status risk compared to TT and TC carriers (OR 0.36; 95% CI 0.19–0.65; P = 0.0007) (Table 4). The T alleles appear to decrease the genetic susceptibility to a lower transport status compared to C alleles.

**Table 4:** rs3025239 genotype in the H/HA and L/LA groups

SNP	Genotype	H/HA Group n = 94	L/LA Group n = 106	X^2^	P value
rs3025039	TT + TC	25	53	11.47	***0.0007***
	CC	69	53		

As shown in Table 5, there were no significant differences in age\BMI\Hb\Alb\CRP\UF\urine between rs3025039 polymorphisms. A total of 21.79% of CC carriers had diabetes, while the rate of TT or TC carriers was 15.57%. Patients with TT/CT had a higher Kt/V than those with the CC genotype.

**Table 5:** Association between clinical characteristics and rs3025039 genotype

SNP	rs3025039			
**Genotype**	**TT + TC** **n = 122**	**CC** **n = 78**		**P value**
Age (year)	53.95 ± 15.80	50.97 ± 13.55		0.156
BMI (kg/m2)	21.62(19.59,24.00	20.84(19.54,23.55)		0.383
Diabetes (%)	19(15.57)	17(21.79)		0.265
Hb (g/L)	103.60 ± 18.38	101.50 ± 23.22		0.502
ALB (g/L)	35.80(32.18,39.43)	36.30(33.25,38.83)		0.915
CRP (mg/L)	2.41(0.89,4.89)	2.92(0.98,3.83)		0.844
UF (ml)	128.70 ± 563.80	83.51 ± 582.20		0.588
Urine (ml)	1033.00 ± 610.60	1104.00 ± 661.20		0.446
4 h D/P Cr	0.61 ± 0.11	0.65 ± 0.12		***0.029***
Kt/V	2.25(1.88,2.73)	2.03(1.77,2.48)		***0.016***
nPCR [g/(kg·d)]	0.85(0.78,1.08)	0.92(0.77,1.13)		0.132

BMI: body mass index; Hb: haemoglobin; Alb: serum albumin; CRP: C reactive protein; UF: ultrafiltration; 4 h D/P Cr: 4-hour dialysate over plasma ratio for creatinine; Kt/V: urea clearance index; nPCR: normalized protein catabolic rate

### Association between KDR polymorphisms and peritoneal transport status

We also examined the association between KDR polymorphisms and the 4 h D/P Cr. The allelic distributions were all in conformity with Hardy-Weinberg equilibrium.

**Fig. 3:**
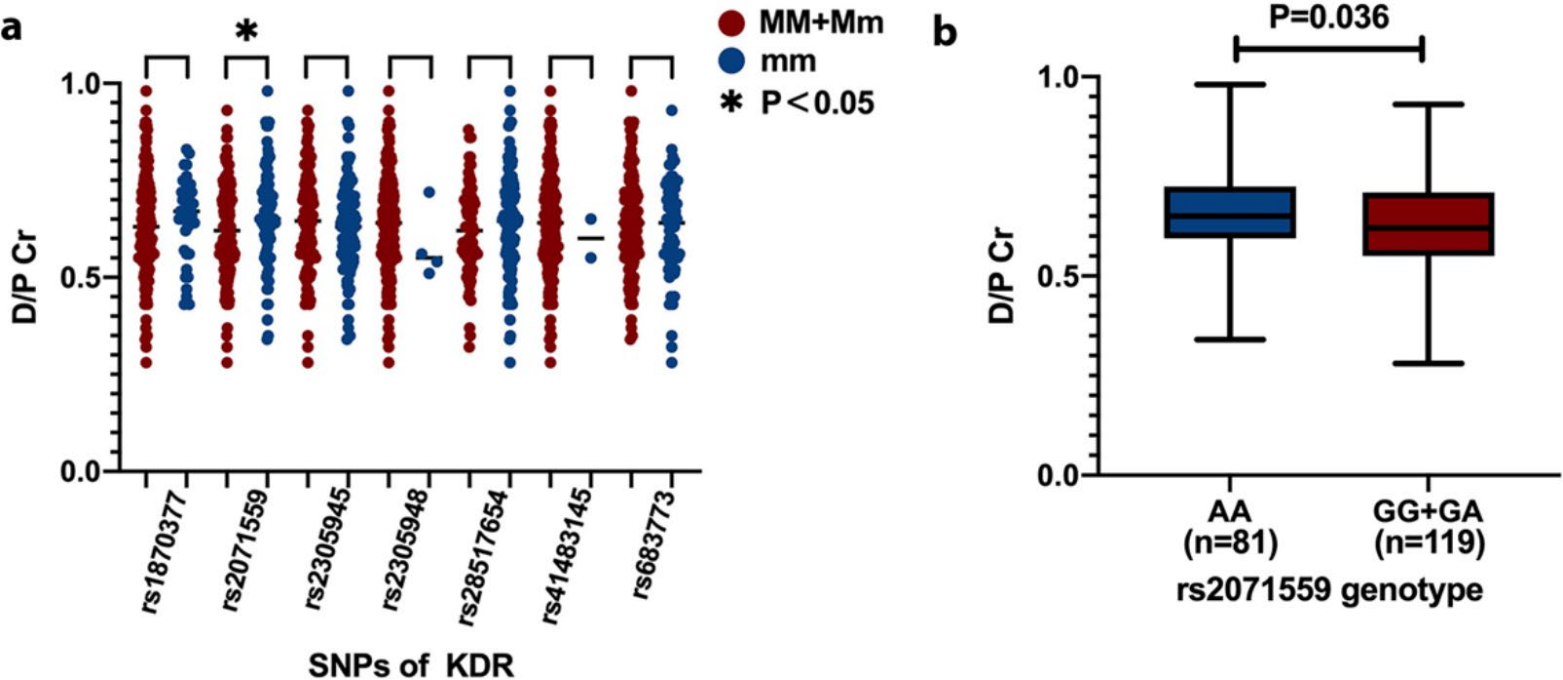
(a) KDR polymorphisms and D/P Cr. M: alteration allele; m: reference allele. (b) *rs2071559 genotypes and D/P Cr*

Among the seven selected tagSNPs in the KDR gene, rs2071559 was shown to be associated with the 4 h D/P Cr, while the other six SNPs in rs1870377, rs2305945, rs2305948, rs28517645, rs41483145 and rs683773 had no effect on the 4 h D/P Cr (Fig. 3a).

Patients carrying two minor alleles at rs2071559 (AA genotype) had a significantly higher D/P Cr than those carrying the GG or GA genotype (AA vs. GG + GA: 0.65 ± 0.12 vs. 0.62 ± 0.12; P = 0.036) (Fig. 3b).

Furthermore, H/HA transport status patients had a remarkably higher frequency of the AA genotype than L/LA transport status patients (OR 0.56; 95% CI 0.31–0.99; P = 0.045) (Table 6). The G allele was shown to be associated with an increased risk of L/LA transport status compared to the A allele.

**Table 6:** rs2071559 genotype in the H/HA and L/LA groups

SNP	Genotype	H/HA Group n = 94	L/LA Group n = 106	X^2^	P value
rs2071559	GG + GA	49	70	4	***0.045***
	AA	45	36		

There were no significant differences between the GG + GA and AA genotypes of rs2071559 in age, BMI, Hb, Alb, CRP, UF or urine volume. A total of 23.46% of AA carriers had diabetes, which was higher than the proportion of GG or GA carriers (14.29%). However, AA carriers had a significantly higher Kt/V than GG and GA carriers (Table 7).

**Table 7:** Association between clinical characteristics and rs2071559 genotype

SNP	rs2071559			
**Genotype**	**GG + GA** **n = 119**	**AA** **n = 81**		**P value**
Age (year)	52.45 ± 14.47	51.65 ± 14.63		0.703
BMI (kg/m2)	21.56(19.59,24.57)	20.70(19.52,23.01)		0.272
Diabetes (%)	17(14.29)	19(23.46)		0.132
Hb (g/L)	101.80 ± 20.60	103.10 ± 22.72		0.655
ALB (g/L)	36.10(33.90,39.10)	35.70(31.95,39.15)		0.384
CRP (mg/L)	2.65(0.61,5.18)	3.00(1.00,3.74)		0.641
UF (ml)	105.20 ± 536.80	95.23 ± 628.30		0.904
Urine (ml)	1016.00 ± 640.00	1165.00 ± 636.80		0.107
4 h D/P Cr	0.62 ± 0.12	0.65 ± 0.12		***0.036***
Kt/V	2.07(1.78,2.41)	2.24(1.87,2.71)		***0.041***
nPCR [g/(kg·d)]	0.92(0.79,1.08)	0.91(0.75,1.16)		0.713

BMI: body mass index; Hb: haemoglobin; Alb: serum albumin; CRP: C reactive protein; UF: ultrafiltration; 4 h D/P Cr: 4-hour dialysate over plasma ratio for creatinine; Kt/V: urea clearance index; nPCR: normalized protein catabolic rate

## Discussion

In this study, we analysed the association between baseline PSTR and genetic polymorphisms of two genes (VEGF and KDR) in a Chinese Han population. The results showed that SNPs of rs3025039 in VEGF and SNPs of rs2071559 in KDR were significantly associated with initial 4 h D/P Cr. Genetic factors related to neovascularization are related to the initial PSTR in PD.

Fast initial peritoneal transport status is an independent risk factor for long-term prognosis in patients with PD. Meta-analysis showed that for every 0.1 increase in the dialysate over plasma ratio for creatinine (D/P Cr), the relative risk of death increased by 1.15-fold, which was equivalent to a 21.9% increase in low average transporters, a 45.7% increase in high average transporters and a 77.3% increase in high transport of the patients compared with the low transport patients. For every 0.1 increase in the D/P Cr, the risk of death associated with technology failure increased by 1.18-fold [22].

The pathophysiological mechanism of late acquired high transport induced by long-term peritoneal dialysis is different from that of early inherent high transport [23, 24]. After initiating PD, significant changes occur in the transport characteristics, which may be due to the differences in the structure and function of the peritoneum before dialysis; these differences mainly manifest as microvascular endothelial function and microinflammation of the peritoneum [11, 25]. Genetic factors are involved in determining initial peritoneal status. Previous studies in our centre have shown that the gene polymorphisms of vascular-related TIE2(rs639225) and inflammation-related IL-6(rs13306345) are associated with high initial peritoneal transport [26].

The VEGF gene is located on chromosome 6p21.3, containing and contains 8 exons and 7 introns. It belongs to the VEGF/platelet-derived growth factor gene family, also known as the growth factor cystine superfamily [27]. VEGF is an important regulatory factor in endothelial cell physiology and a major specific growth factor of endothelial cells [28, 29]. VEGF affects the inflammatory environment by acting as a proinflammatory cytokine through its ability to act as a monocyte chemotactic agent [30].

To date, VEGF gene polymorphisms have been confirmed to be associated with a variety of diseases by participating in angiogenesis. The rs3025039 polymorphism was found to be associated with elevated plasma VEGF levels in glioma and many other cancers [14–17, 29, 31].

The biological function of VEGF is achieved through its receptor, mainly for KDR [13]. The KDR gene is located in chromosomal region 4q11-q12 and contains 26 exons [27]. It is mainly expressed in vascular endothelial cells and lymphatic vessels and is the main receptor in the angiogenesis signalling pathway. The gene polymorphisms of rs2071559 were also reported to be associated with tumour recurrence [20].

The two SNPs rs3025039 of VEGF and rs2071559 of KDR, which were found to be associated with initial higher peritoneal transport status, are located in the 3’UTR and upstream, which belong to the noncoding region. Although this region cannot encode proteins, it is indispensable for the expression of genetic information. The nucleotide sequences can regulate the expression of genetic information to have genetic effects. Genetic variation due to gene polymorphisms in noncoding regions may partially explain the significant association between SNPs of the two tested genes and initial peritoneal high transport risk. ESRD patients with the CC genotype of rs3025039 in VEGF or the AA genotype of rs2071559 in KDR are more likely to have a congenital high transport type that may be associated with a poor outcome. Such patients may require a more comprehensive evaluation before dialysis, and perhaps haemodialysis or early intervention through peritoneal dialysis may improve the outcome.

This study also has some limitations. First, our study was conducted in a single population, so our results may not be applicable to other populations due to genetic variation. Second, our study is a single-centre study with a small sample size. Therefore, in future research, we will include patients from multiple centres, increase the sample size, and try to measure the concentration of VEGF and KDR in the initial peritoneum. Tag-SNPs may not completely cover all the genetic variants, and some existing SNPs of VEGF or KDR were not included. Furthermore, the coreceptor neuropilin-1 (Nrp-1) can enhance the effect of VEGF binding to KDR, which plays an important role in the mesothelial to mesenchymal transition (MMT) in long-term PD and causes peritoneal membrane dysfunction [32]. In future studies, we will further study the SNPs of Nrp-1 and focus on the corresponding pathways of SNPs.

At present, the gene polymorphisms of VEGF and KDR are mainly used in the field of cancer. In this study, we investigated for the first time the genetic association between the initial PD transport status and VEGF/KDR. The CC genotype of rs3025039(VEGF) and AA genotype of rs2071559(KDR) could be predictors of initial high transport status, and allele T of rs3025039 or allele G of rs2071559 were associated with the occurrence of initial lower transport status. These results suggest that VEGF and KDR may be used as genetic markers to identify the initial fast PSTR.

## Conclusion

The baseline PSTR was partly determined by VEGF and KDR gene polymorphisms. The CC genotype of rs3025039(VEGF) and AA genotype of rs2071559(KDR) could be predictors of initial high transport status.

## Data Availability

The datasets during and/or analysed during the current study available from the corresponding author on reasonable request.

## List of abbreviations

PD: Peritoneal dialysis
PSTR: Peritoneal solute transfer rate
VEGF: Vascular endothelial growth factor
KDR: Kinase insert domain containing receptor
ESRD: End-stage renal disease
SNP: Single nucleotide polymorphism
PET: Peritoneal equilibration test
4 h D/P Cr: 4-hour dialysate-to-plasma ratio for creatinine
CHB&CHS: Han Chinese in Beijing & Southern Han Chinese
MAF: Minor allele frequency
HWE: Hardy Weinberg equilibrium
PCR: Polymerase chain reaction
H: High transport status
HA: High average transport status
LA: Low average transport status
L: Low transport status
BMI: Body mass index
Hb: Haemoglobin
Alb: Serum albumin
CRP: C reactive protein
UF: Ultrafiltration
Kt/V: Urea clearance index
nPCR: Normalized protein catabolic rate
